# Regulating interaction with surface ligands on Au_25_ nanoclusters by multivariate metal–organic framework hosts for boosting catalysis

**DOI:** 10.1093/nsr/nwae252

**Published:** 2024-07-23

**Authors:** He Wang, Xiaokang Liu, Yulong Zhao, Zhihu Sun, Yue Lin, Tao Yao, Hai-Long Jiang

**Affiliations:** Hefei National Research Center for Physical Sciences at the Microscale, University of Science and Technology of China, Hefei 230026, China; Department of Chemistry, University of Science and Technology of China, Hefei 230026, China; National Synchrotron Radiation Laboratory, University of Science and Technology of China, Hefei 230029, China; Hefei National Research Center for Physical Sciences at the Microscale, University of Science and Technology of China, Hefei 230026, China; Department of Chemistry, University of Science and Technology of China, Hefei 230026, China; National Synchrotron Radiation Laboratory, University of Science and Technology of China, Hefei 230029, China; Hefei National Research Center for Physical Sciences at the Microscale, University of Science and Technology of China, Hefei 230026, China; National Synchrotron Radiation Laboratory, University of Science and Technology of China, Hefei 230029, China; Hefei National Research Center for Physical Sciences at the Microscale, University of Science and Technology of China, Hefei 230026, China; Department of Chemistry, University of Science and Technology of China, Hefei 230026, China

**Keywords:** metal nanoclusters, metal–organic frameworks, heterogeneous catalysis, microenvironment modulation

## Abstract

While atomically precise metal nanoclusters (NCs) with unique structures and reactivity are very promising in catalysis, the spatial resistance caused by the surface ligands and structural instability poses significant challenges. In this work, Au_25_(Cys)_18_ NCs are encapsulated in multivariate metal–organic frameworks (MOFs) to afford Au_25_@M-MOF-74 (M = Zn, Ni, Co, Mg). By the MOF confinement, the Au_25_ NCs showcase highly enhanced activity and stability in the intramolecular cascade reaction of 2-nitrobenzonitrile. Notably, the interaction between the metal nodes in M-MOF-74 and Au_25_(Cys)_18_ is able to suppress the free vibration of the surface ligands on the Au_25_ NCs and thereby improve the accessibility of Au sites; meanwhile, the stronger interactions lead to higher electron density and core expansion within Au_25_(Cys)_18_. As a result, the activity exhibits the trend of Au_25_@Ni-MOF-74 > Au_25_@Co-MOF-74 > Au_25_@Zn-MOF-74 > Au_25_@Mg-MOF-74, highlighting the crucial roles of microenvironment modulation around the Au_25_ NCs by interaction between the surface ligands and MOF hosts.

## Teaser

Regulating surface ligands on metal nanoclusters by MOF hosts improves single electron transfer and metal site accessibility for enhanced catalysis.

## INTRODUCTION

The design and fabrication of structurally precise metal sites are crucial for the development of high-performance heterogeneous catalysts [1–3]. Metal nanoclusters (NCs) with atomic-level structural precision have attracted intense attention due to their small size, uniform active sites and unique electronic structures, giving rise to outstanding activity and selectivity in various catalytic reactions [4–7]. Unfortunately, the surface of metal NCs is always covered with numerous organic ligands that substantially influence catalysis. On the one hand, these surface ligands are detrimental to the accessibility of metal sites [8,9]; on the other, they optimize the electronic structure of metal NCs and improve the activity and selectivity of specific reactions [10–12]. Therefore, the role of surface ligands is akin to a double-edged sword affecting the catalytic properties [13]. In this context, it would be of great importance to preserve the strength yet suppress the drawback of surface ligands on metal NCs, so as to promote the catalysis of metal NCs. Inspired by the crucial roles of the surrounding microenvironment around catalytic centers in bio-enzyme catalysis [14], through the manipulation of the organic surface ligands on metal NCs, it might be possible to improve the substrate accessibility to and electronic structure of catalytic metal sites. To this end, it would be highly desired to develop porous hosts featuring well-defined structures and tunable interaction with the surface ligands. This would allow the regulation of their configuration and movement, thereby influencing the accessibility and activity of central metal sites.

To achieve the aforementioned goal, metal–organic frameworks (MOFs)—a class of crystalline porous materials with coordinatively unsaturated metal sites (for interacting with surface ligands) and tunable pore sizes (for hosting metal NCs)—would be promising candidates [15–18]. MOFs have been demonstrated to be very suitable for incorporating diverse catalytically active species, including single-atom metal sites [19,20], metal nanoparticles [21,22], organic molecules [23], enzymes [24,25], etc., into pore spaces for enhanced catalysis. There have been also a couple of reports on the integration of metal NCs with MOFs in catalysis [26–31], where the regulation of surface ligands on metal NCs has not yet been investigated, except for ligand removal in the only study [32]. Given that the structure and properties of metal NCs are particularly sensitive to the surrounding microenvironment [33], it would be reasonable to adopt MOFs to modulate the microenvironment around metal NCs by regulating their surface ligands based on their interaction with MOFs for enhanced catalytic performance [34]. It is expected that the interactions between surface ligands of metal NCs and MOFs would effectively suppress the free vibration of surface ligands in solution and reduce their hindrance of substrate access to the metal sites [26]. Moreover, the spatial confinement effect of MOFs would significantly improve the catalytic stability of metal NCs [27–29]. However, to our knowledge, it remains unknown how the interaction with porous hosts (e.g. MOFs) regulates the surface ligands on metal NCs for improved catalysis.

**Figure 1.**
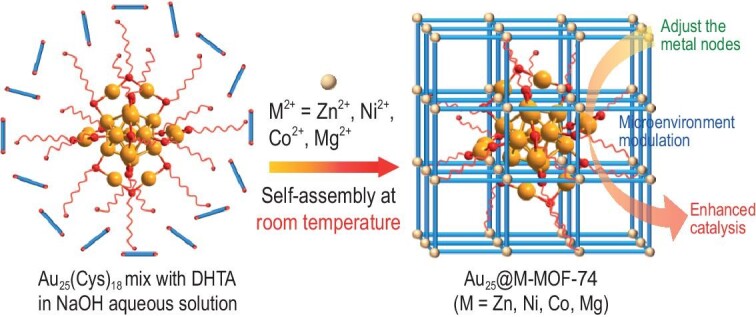
Illustration showing the synthetic route to Au_25_@M-MOF-74 (M = Zn, Ni, Co, Mg) for enhanced catalysis through microenvironment modulation around Au_25_(Cys)_18_ by adjusting the metal nodes on MOF pore walls.

In this work, Au_25_(Cys)_18_ NCs are encapsulated into representative multivariate MOFs, M-MOF-74 (M = Zn, Ni, Co, Mg), based on coordinated self-assembly and electrostatic interactions to afford Au_25_@M-MOF-74 (Fig. 1). Significant differences in the fluorescence properties of Au_25_(Cys)_18_ are observed among these materials, which can be attributed to the restricted influence of M-MOF-74 encapsulation on the surface-ligand vibration behavior of Au_25_(Cys)_18_. Accordingly, the encapsulation of Au_25_ NCs in M-MOF-74 significantly improves its catalytic efficiency in the intramolecular cascade reaction of 2-nitrophenyl cyanide. Furthermore, X-ray absorption spectroscopy (XAS) results indicate that the electron density of Au_25_(Cys)_18_ and the length of the Au–Au bonds within the core of Au_25_(Cys)_18_ can be systematically regulated in addition to the interaction strengths between M-MOF-74 and Au_25_(Cys)_18_ NCs. Such interaction modulates the microenvironment of Au_25_(Cys)_18_, which improves the Au_25_ accessibility and facilitates the electron transfer from the Au_25_ NCs to substrates responsible for promoting the catalysis. As a result, the catalytic activity can be further regulated by doping the metal nodes of MOF-74 in the following sequence: Ni > Co > Zn > Mg. Moreover, Au_25_@Ni-MOF-74 exhibits far superior catalytic activity and stability to the control of Au_25_/Ni-MOF-74 that is made by supporting the Au_25_ NCs on the outer surface of Ni-MOF-74. As far as we know, this is the first report on regulating interaction by MOFs with surface ligands of metal clusters, resulting in enhanced catalysis.

## RESULTS AND DISCUSSION

### Synthesis and characterization of the Au_25_@Ni-MOF-74 catalyst

The Au_25_(Cys)_18_ NCs were selected due to their relatively stable structure and abundance of carbonyl groups on the surface (Fig. S1a) [35]. It was synthesized by the NaOH-mediated NaBH_4_ reduction method and the high purity of the synthesized Au_25_(Cys)_18_ was confirmed by using UV–vis spectroscopy and high-angle annular dark-field scanning transmission electron microscopy (HAADF-STEM) (Fig. S2) [35]. To prevent structural damage to Au_25_(Cys)_18_ under high-temperature solution conditions, *in situ* growth of the MOF-74 outer-shell was developed. The MOF linker, 2,5-dihydroxyterephthalic acid (DHTP) and Au_25_(Cys)_18_ were pretreated in aqueous NaOH solution, inducing the deprotonation and subsequently promoting the dissolution of DHTP in water. This facilitates the reaction with metal ions, leading to the growth of MOF at room temperature, and promotes the dispersion and encapsulation of Au_25_(Cys)_18_ in MOF-74. Subsequently, the metal ions underwent coordination self-assembly with Au_25_(Cys)_18_ and DHTP at room temperature, resulting in the formation of Au_25_@MOF-74 [36]. Taking advantage of the multivariate feature of MOFs, M-MOF-74 (M = Ni, Co, Mg) with Ni^2+^, Co^2+^ or Mg^2+^ in the Zn-oxo chains was obtained (Fig. S1b), ensuring their similar crystallinity and sizes, by mixing these metal acetates together with zinc acetate in the synthesis of Zn-MOF-74. Accordingly, Au_25_@M-MOF-74 (M = Zn, Ni, Co, Mg) were fabricated following a similar synthetic route to Au_25_@MOF-74, which would serve as an ideal platform for regulating the interaction with the surface ligands of Au_25_(Cys)_18_. In addition, due to the presence of a large number of negatively charged carboxyl groups on the surface, Au_25_(Cys)_18_ can be attached to the outer surface of Ni-MOF-74 by electrostatic interaction to yield Au_25_(Cys)_18_/Ni-MOF-74 [30,31].

Powder X-ray diffraction (XRD) patterns indicate that the M-MOF-74 has similar crystallinity and Au_25_(Cys)_18_ encapsulation or support has no influence on the structural integrity and crystallinity of the MOFs (Fig. S3). Nitrogen sorption results demonstrate that Au_25_@M-MOF-74 and Au_25_/Ni-MOF-74 maintain the high porosity and very similar pore size distribution, indicating that the introduction of Au_25_(Cys)_18_ does not affect the MOF microporous structure (Fig. S4). The Brunauer-Emmett-Teller surface area of Au_25_@Ni-MOF-74 is 639 m^2^/g, similar to that of Au_25_@Zn-MOF-74 (604 m^2^/g), Au_25_@Co-MOF-74 (609 m^2^/g) and Au_25_@Mg-MOF-74 (610 m^2^/g), reflecting that different metal nodes have little influence on the surface area. Due to the pore space occupation of MOF-74 by the Au_25_ NCs, their surface areas are reasonably lower than Au_25_/MOF-74 (667 m^2^/g). For Au_25_@M-MOF-74, Au_25_(Cys)_18_ can be isolated by the removal of MOF-74 using dilute hydrochloric acid. UV–vis spectra for the isolated Au_25_(Cys)_18_ NCs, upon removing the MOF, showcase the characteristic bands (Fig. S5), demonstrating that MOF-74 encapsulation does not influence the integrity of the Au_25_(Cys)_18_ structure.

**Figure 2.**
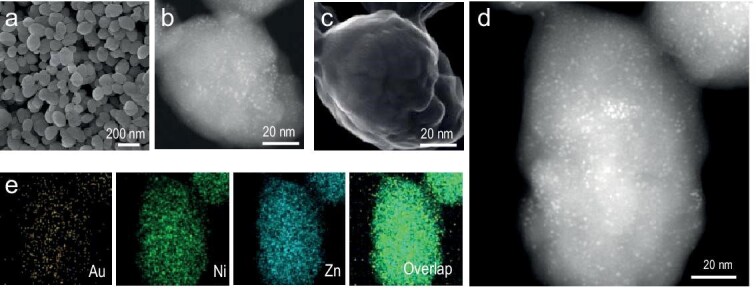
(a) SEM image, (b) HAADF-STEM and (c) the corresponding SE-STEM image of Au_25_@Ni-MOF-74. (d) HAADF-STEM image and (e) corresponding EDS elemental mapping of Au, Ni and Zn, and their overlap for Au_25_@Ni-MOF-74.

Scanning electron microscopy (SEM) images show that Au_25_@M-MOF-74 and Au_25_/Ni-MOF-74 have similar particle sizes and morphology (Fig. 2a and Fig. S6). From the high-angle annular dark-field-scanning transmission electron microscopy (HAADF-STEM) images, it can be observed that Au_25_(Cys)_18_ NCs are uniformly dispersed throughout the Ni-MOF-74 support (Fig. 2b and Fig. S7a). Direct comparison of the HAADF-STEM and secondary electron STEM (SE-STEM) images acquired at the same location provides direct evidence that Au_25_(Cys)_18_ NCs are encapsulated inside Ni-MOF-74 in Au_25_@Ni-MOF-74, as indicated by unobservable Au_25_ NCs in the SE-STEM image (Fig. 2b and c). By comparison, the Au_25_ NCs can be clearly observed in both images, showing that the Au_25_ NCs are supported on the surface of Ni-MOF-74 in Au_25_/Ni-MOF-74 (Fig. S8). HAADF-STEM images of Au_25_@Ni-MOF-74 projected at different tilt angles (from +30° to −30°) show that the Au_25_ NCs remain monodispersed in the Ni-MOF-74 (Fig. S9). The HAADF-STEM images and corresponding energy dispersive X-ray spectroscopy (EDS) mapping analyses reveal that both metals in M-MOF-74 and Au_25_(Cys)_18_ are uniformly distributed throughout the MOF particle (Fig. 2d and e, and Figs S9–S12). The Au loading amount in Au_25_@M-MOF-74 is controlled to be ∼2 wt% and the mixed metal molar ratio in M-MOF-74 is also maintained at ∼1 according to inductively coupled plasma atomic emission spectroscopy (ICP-AES) results (Table S1).

### Performance of catalytic reduction of 2-nitrobenzonitrile

Given the unique surface and electronic structure of Au_25_(Cys)_18_ NCs, they can serve as electron mediators to initiate catalysis via single electron transfer [37,38]. Therefore, the reduction reaction of 2-nitrobenzonitrile containing electron-transfer processes is adopted, which is an important route for the production of significant pharmaceuticals precursor 2-amniobenzamide [37]. Control experiments indicate that no product is detected in the absence of catalysts or with the use of the four M-MOF-74 catalysts (Table S2). When adopting Au_25_(Cys)_18_ NCs, the activity and selectivity are 20.6% and 91.0%, respectively, indicating that the Au_25_ NCs are able to behave as active species. Unfortunately, they are prone to aggregation, as supported by the UV–vis spectrum and HAADF-STEM observation (Fig. S13). Moreover, no obvious difference in catalytic activity is observed when the same amount of Au_25_ NCs is physically mixed with the four M-MOF-74, respectively, indicating that M-MOF-74 does not affect the catalytic reaction process (Table S2).

**Figure 3.**
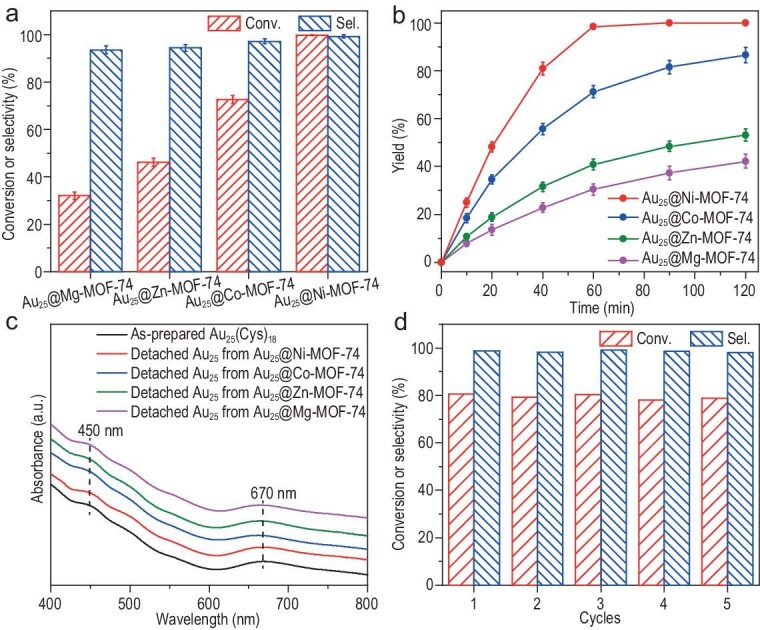
(a) The conversion and selectivity of Au_25_@M-MOF-74 (∼2 wt% Au loading) in the intramolecular cascade reaction of 2-nitrobenzonitrile at 25°C for 1 h. (b) Time-dependent yield of 2-aminobenzamide with Au_25_@M-MOF-74. (c) UV–vis spectra of as-prepared Au_25_(Cys)_18_ and the Au_25_(Cys)_18_ NCs detached from Au_25_@M-MOF-74 after the catalytic reaction. (d) The recyclability tests of Au_25_@Ni-MOF-74.

Strikingly, the encapsulation of Au_25_(Cys)_18_ NCs in M-MOF-74 significantly improves the activity, in which the conversion and selectivity reach 99.8% and 99.2%, respectively, for Au_25_@Ni-MOF-74 (Fig. 3a). In comparison, the conversion and selectivity of Au_25_@Co-MOF-74, Au_25_@Zn-MOF-74 and Au_25_@Mg-MOF-74 are decreased, with conversion of 72.7%, 46.2% and 32.1%, and selectivity of 97.1%, 94.3% and 93.5%, respectively (Fig. 3a). The reaction yield gradually increases along with reaction time, showcasing activity in the order of Au_25_@Ni-MOF-74 > Au_25_@Co-MOF-74 > Au_25_@Zn-MOF-74 > Au_25_@Mg-MOF-74 (Fig. 3b). The results indicate that the metal doping in M-MOF-74 exerts a pivotal influence on the catalytic efficiency of Au_25_(Cys)_18_. Powder XRD patterns of Au_25_@M-MOF-74 do not exhibit any discernible decrease in the MOF crystallinity after the catalytic reaction (Fig. S14); the UV–vis spectra of the detached Au_25_ NCs upon the MOF removal exhibit no substantial alterations with the as-synthesized Au_25_ NCs, effectively demonstrating the structural integrity of both components in Au_25_@M-MOF-74 during the catalytic reaction (Fig. 3c). As a control, the Au nanoparticles (NPs) with surface ligands of Cys were synthesized; the UV–vis spectrum displays a characteristic surface plasmon resonance peak at 520 nm and the HAADF-STEM image indicates that the Au NPs are ∼2 nm in size (Fig. S15). Afterward, they were encapsulated into Ni-MOF-74 to obtain Au_NPs_@Ni-MOF-74 with retained Au sizes and good MOF crystallinity (Fig. S16) via the same coordination self-assembly as that of Au_25_@Ni-MOF-74. The conversion and selectivity of Au_NPs_@Ni-MOF-74 in the reaction are only 15.2% and 89.5%, respectively (Table S2). This is possibly attributed to the fact that the charge-transfer effect on the surface of Au NPs is too weak to efficiently facilitate the migration of electrons from NaBH_4_ to the substrate [37].

The incorporation of Au_25_(Cys)_18_ into M-MOF-74 has been demonstrated to not only influence the activity as noted above, but also strongly improve the catalytic stability. The catalytic yield of Au_25_@Ni-MOF-74 is maintained at 99% for 1 h in the three cycles; in contrast, the yield of Au_25_/Ni-MOF-74 gives 25% in the first cycle and decreases continuously during the next two cycles (Fig. S17), possibly due to the unexpected leaching or aggregation of Au_25_(Cys)_18_. HAADF-STEM images show that the size and dispersion of Au_25_(Cys)_18_ in Au_25_@Ni-MOF-74 remain unchanged after three reaction cycles due to the confined protection by the MOF, whereas significant agglomeration of Au_25_ NCs is observed in Au_25_/Ni-MOF-74 (Fig. S18). No noticeable change occurs in the Au content in Au_25_@Ni-MOF-74 after the reaction; however, a decrease of ∼10% can be found for Au_25_/Ni-MOF-74 (Table S1). These results exemplify the much enhanced stability of Au_25_(Cys)_18_ by MOF encapsulation.

The stability and recyclability of Au_25_@Ni-MOF-74 have been further demonstrated. The activity and selectivity exhibit no significant decrease in the five consecutive cycles with a controlled reaction time of 40 min and ∼80% conversion (Fig. 3d). Powder XRD patterns, HAADF-STEM images and corresponding EDS elemental mapping analyses suggest that the catalyst microstructure can be retained after five consecutive cycles (Figs S19 and S20). In addition, the results of the hot filtration experiment for Au_25_@Ni-MOF-74 manifest that no leaching occurs in Au_25_ NCs and the process is truly heterogeneous catalysis (Fig. S21).

### Mechanism of the catalytic reaction

**Figure 4.**
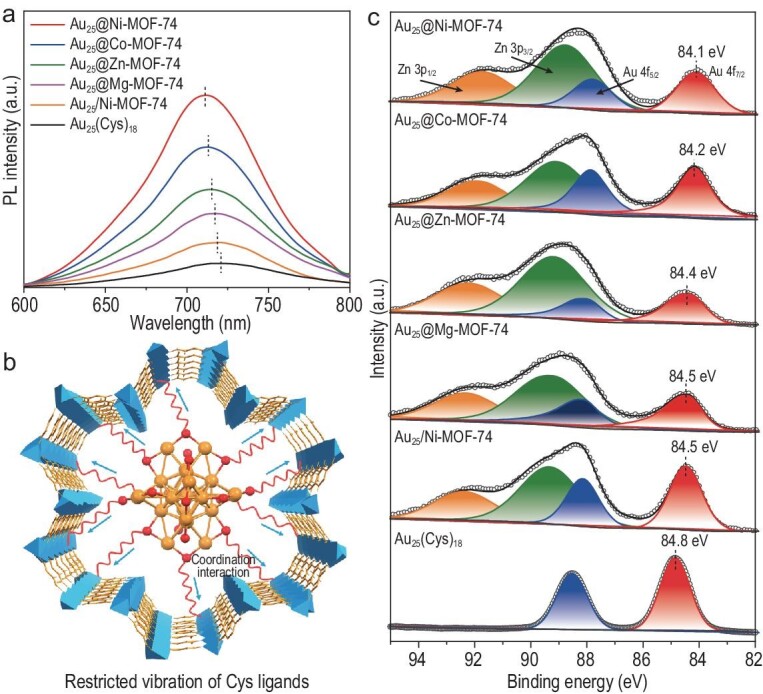
(a) Fluorescence spectra of Au_25_(Cys)_18_, Au_25_@M-MOF-74 and Au_25_@Ni-MOF-74 in aqueous solution. (b) A schematic diagram of encapsulating Au_25_(Cys)_18_ by M-MOF-74 restricts Cys ligand vibrations on its surface. (c) The Au 4f XPS spectra of Au_25_(Cys)_18_, Au_25_/Ni-MOF-74 and Au_25_@M-MOF-74.

Given the significant differences in the activity of Au_25_@M-MOF-74, relevant investigations have been conducted to understand the intrinsic mechanisms involved. The variations in fluorescence emission intensity and wavelength can reflect the extent of motion associated with the vibration and rotation of the ligands on the surface of the metal NCs [39,40]. Under excitation of 420 nm, the photoluminescence (PL) spectrum of Au_25_(Cys)_18_ is in the visible region with a maximum value of ∼720 nm (Fig. 4a) and the PL of Au_25_@M-MOF-74 is substantially enhanced in intensity and undergoes an obvious blue shift compared with that of Au_25_(Cys)_18_. The phenomenon can be ascribed to the coordination interactions between the metal-oxo chain in M-MOF-74 and the free carboxyl groups from the Cys on the surface of Au_25_(Cys)_18_, which restricts the vibration and rotation of the Cys ligands and inhibits the non-radiative leaps of Au_25_(Cys)_18_, thereby improving the emission efficiency [41]. Moreover, this interaction affects the electronic structure of Au_25_(Cys)_18_, giving rise to the blue shift of its fluorescence signal [39]. Therefore, the PL intensity and peak shift of Au_25_(Cys)_18_ incorporated in M-MOF-74 can reflect the degree of Cys rigidification (Fig. 4b). The PL intensities and peak shift of Au_25_(Cys)_18_ follow the trend of Au_25_@Ni-MOF-74 > Au_25_@Co-MOF-74 > Au_25_@Zn-MOF-74 > Au_25_@Mg-MOF-74, in agreement with the order of their catalytic activities. This reflects that the difference in the coordination strength between the MOF metal-oxo chains with carboxylate groups on the Au_25_(Cys)_18_ surface modulates the microenvironment of Au_25_(Cys)_18_, influencing the catalytic activity. The PL intensity and shift of Au_25_@M-MOF-74 are apparently greater than those of Au_25_/MOF-74, suggesting that the MOF encapsulation is more favorable for the rigidification of Cys and improves the activity to a larger extent.

X-ray photoelectron spectroscopy (XPS) analysis demonstrates a significant increase in the Au electron density of Au_25_(Cys)_18_ upon integration with M-MOF-74 (Fig. 4c). Interestingly, the electron density of Au in Au_25_@M-MOF-74 is in a sequence that is consistent with their activity order, disclosing the fact that the charge-transfer interaction between Au_25_(Cys)_18_ and M-MOF-74 benefits the activity. In addition, the change in the Au electron density in Au_25_/Ni-MOF-74 is relatively small, further suggesting that Au_25_(Cys)_18_ supported on MOF cannot create a strong interaction with Au_25_(Cys)_18_.

UV–vis spectroscopy and electron spin resonance (ESR) spectroscopy are further adopted to investigate the electron-transfer process in the catalytic reactions. The Au_25_ NCs with different charges have unique UV–vis absorption spectra, which can clearly distinguish the charge states of Au_25_ NCs [42]. When 2-nitrobenzonitrile is added as the substrate to the Au_25_(Cys)_18_ with a negative charge (abbreviated as Au_25_^−^) in the aqueous solution, its UV–vis spectrum displays a distinct change from the characteristic spectrum of uncharged Au_25_(Cys)_18_ (abbreviated as Au_25_^0^). After NaBH_4_ is added, the characteristic absorption peak of Au_25_^−^ reappears (Fig. S22), indicating the recovery of Au_25_^−^. Furthermore, ESR experiments indicate that Au_25_@Ni-MOF-74 and the mixture of Ni-MOF-74 with 2-nitrobenzonitrile do not result in any signal, whereas the mixture of Au_25_(Cys)_18_ or Au_25_@Ni-MOF-74 with 2-nitrobenzonitrile results in a triple peak corresponding to the N radicals (Fig. S23). These results confirm that Au_25_(Cys)_18_ is an electronic mediator that continuously transfers electrons from NaBH_4_ to the substrate. In addition, a range of catalytic experiments with substrate analogs have been performed (Table S3). The functional group of the substrates can be efficiently and completely reduced when the unsaturated group is placed at the ortho position only, suggesting that an intermolecular cascade reaction has occurred. To further investigate the catalytic mechanism, deuterium-labeling experiments are also conducted. When H_2_O is replaced by D_2_O and NaBH_4_ is replaced by NaBD_4_ in the reaction system, the molecular weight of the products detected by using mass spectrometry increases (Fig. S24), implying that H_2_O and NaBH_4_ are involved in the reaction. As a result, reaction paths can be proposed (Fig. S25).

### The geometric structure and electronic properties of Au_25_(Cys)_18_

**Figure 5.**
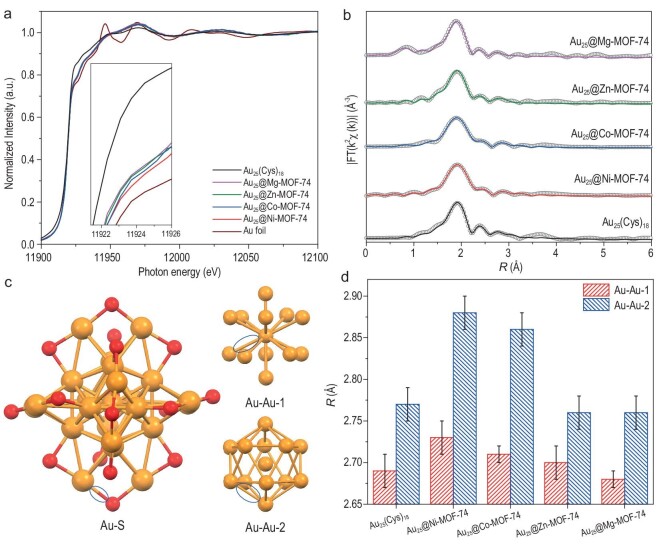
(a) Au L_3_-edge XANES spectra of Au foil, Au_25_(Cys)_18_ and Au_25_@M-MOF-74. (b) Au L_3_-edge FT-EXAFS spectra of Au_25_(Cys)_18_ and Au_25_@M-MOF-74. (c) Attribution of the EXAFS-analysed Au_25_(Cys)_18_ structure to three distinct bond domains (the Au–S, Au–Au-1 and Au–Au-2 highlighted with ellipses). (d) The bond lengths of Au–Au-1 and Au–Au-2 are extracted from the refinement of the FT-EXAFS spectra of Au_25_(Cys)_18_ and Au_25_@M-MOF-74.

Given that the X-ray absorption fine structure (XAFS) is sensitive to the structure of metal NCs [43,44], the Au L_3_-edge XAFS is adopted to investigate the effect of MOF encapsulation on the geometric structure and electronic properties of Au_25_(Cys)_18_. X-ray absorption near edge structure (XANES) spectra display obvious differences between the peak profiles of Au_25_(Cys)_18_ and fcc-structured Au foil (Fig. 5a). Moreover, the peak profile of Au_25_(Cys)_18_ almost remains after its integration with M-MOF-74, suggesting that the structure of Au_25_(Cys)_18_ is preserved in Au_25_@M-MOF-74, which is in agreement with the above results. The white line peak corresponds to the electronic transition from the core level to the unoccupied 5d valence level and the variation in the intensity of the white line peak can drive the absorption edge to different directions of energy, thus explaining different electronic states [45]. In comparison with that of the Au foil, the spectrum of Au_25_(Cys)_18_ exhibits a more intense white line peak at ∼11 924 eV, which is due to the electron-withdrawing property of the thiol ligand and the reduced 5d electron density of surface Au atoms. On the other hand, the white line intensity of Au_25_@M-MOF-74 is lower than that of Au_25_(Cys)_18_, suggesting the greater occupation of the 5d electronic state in Au_25_@M-MOF-74. This is likely due to the MOF’s constraining thiol ligand vibrations on the Au_25_(Cys)_18_ surface, thereby weakening the electron-withdrawing effect of the thiol ligand toward the Au atoms. In addition, the intensity order of the white line peaks in Au_25_@M-MOF-74 is further demonstrated by means of differential spectroscopy of XAFS, which is consistent with the results of XPS analysis (Fig. S26), confirming that the microenvironment modulation caused by the interaction between M-MOF-74 and the thiol ligand regulates the Au_25_ electronic structure.

To quantify the local atomic structure of Au_25_(Cys)_18_ in MOF-74, the extended X-ray absorption fine structure (EXAFS) region has been analysed. All Au_25_@M-MOF-74 samples exhibit oscillation patterns that are similar to those of Au_25_(Cys)_18_, whereas the oscillation intensity increases, suggesting that the surrounding microenvironment modulation driven by the MOF encapsulation can affect the degree of Au_25_(Cys)_18_ disorder (Fig. S27). Fourier transform (FT) is performed in the R-space of the EXAFS spectra for Au_25_(Cys)_18_ and Au_25_@M-MOF-74 (Fig. 5b). For Au_25_(Cys)_18_, three prominent features are shown at 1.9, 2.4 and 2.7 Å; thus, the fittings are performed using three coordination paths. As previously reported [46], the first peak represents the S atom directly coordinated to Au, i.e. Au–S on the motif of Au_25_(Cys)_18_, and the other two peaks represent contributions from two Au–Au pathways (Fig. 5c). By comparing the curve-fitting analysis of Au_25_(Cys)_18_ and Au_25_@M-MOF-74 EXAFS results (Table S4), it is observed that the bond length of Au–S is very similar (Fig. S28), while Au–Au-1 and Au–Au-2 increase when Au_25_(Cys)_18_ NCs are encapsulated in Ni-MOF-74 and Co-MOF-74, suggesting that the Au_25_(Cys)_18_ nucleus undergoes expansion in response to the stronger interaction with M-MOF-74 (Fig. 5d). Notably, the Au–Au bond length sequence in Au_25_@M-MOF-74 is in good agreement with their catalytic activity, suggesting that the catalytic performance of metal NCs can be optimized by manipulating the metal-oxo chains in M-MOF-74 hosts. Furthermore, the Au L_3_-edge XANES and EXAFS spectra of Au_25_@Ni-MOF-74 after the reaction are similar to those before the reaction (Fig. S29), further supporting that the structure of the Au_25_ NCs in Au_25_@Ni-MOF-74 barely changes after the catalytic reaction, matching the UV–vis spectra above (Fig. 3c) and demonstrating that MOF encapsulation improves the structural stability of metal NCs.

Based on the above analysis, it can be concluded that the strong coordination interaction between M-MOF-74 and Au_25_(Cys)_18_ results in the rigidification of surface ligands and the expansion of the Au nucleus. Given that ligand vibrations on the surface of Au_25_ NCs in the reaction solution prevent the substrate from accessing the Au sites, rigidifying the surface ligands and expanding the gold nucleus are expected to increase the available spatial domain for substrate accessibility, thereby boosting activity. Additionally, the different interaction between tunable M-MOF-74 and surface ligands of Au_25_(Cys)_18_ effectively regulates the electron transfer from the MOF to Au_25_ NCs. Due to the electrophilicity of the nitro and cyano groups in 2-nitrobenzonitrile, metal surfaces with higher electron densities will give stronger interactions with the substrate [47,48]. Overall, optimization of the accessibility and electron density of the Au_25_ NCs facilitates electron transfer with the substrate and promotes the conversion [49].

## CONCLUSION

In summary, atomically precise Au_25_(Cys)_18_ NCs have been successfully encapsulated in M-MOF-74 with different metal nodes through coordination self-assembly, yielding Au_25_@M-MOF-74 composites for the intramolecular cascade reaction of 2-nitrobenzonitrile. Strikingly, the activity and stability of Au_25_(Cys)_18_ are significantly enhanced upon being incorporated into MOF-74, surpassing those of Au_25_/MOF-74. Remarkably, doping different metal species into the metal-oxo chains in MOF-74 showcases significant activity difference in the order of Au_25_@Ni-MOF-74 > Au_25_@Co-MOF-74 > Au_25_@Zn-MOF-74 > Au_25_@Mg-MOF-74. Both fluorescence and XAFS analyses demonstrate that the engineering of metal-oxo nodes in MOFs gives rise to the rigidification of surface ligands on Au_25_(Cys)_18_ and induces expansion at the Au nucleus level, improving the accessibility of the Au sites. Moreover, the stronger coordination interaction between M-MOF-74 and Au_25_(Cys)_18_ further increases the electron density of Au_25_ NCs, which is favorable to the substrate activation, leading to enhanced activity. This work describes improved single electron transfer and metal site accessibility of metal NCs by regulating the interaction between their surface ligands and multivariate MOF hosts, which opens a new avenue for boosting the catalysis of metal NCs by surface microenvironment modulation.

## MERHODS

### Synthesis of Au_25_@Zn-MOF-74

Typically, 0.2 mL of aqueous solution of Au_25_(Cys)_18_ (15 mg/mL) was added to 1 mL of 0.73 M NaOH aqueous solution of DHTP (0.183 mmol) at 25°C. Then, 1 mL of aqueous solution of Zn(CH_3_COO)_2_·2H_2_O (0.732 mmol) was mixed with the solution, sonicated for 1 min and stirred for 6 h at 25°C. The precipitate was collected by centrifugation and washed with H_2_O and MeOH three times. Finally, the precipitate was soaked in MeOH for 48 h and dried under a vacuum at 60°C overnight.

### Synthesis of Au_25_@M-MOF-74 (M = Ni, Co, Mg)

Typically, 0.2 mL of Au_25_(Cys)_18_ aqueous solution (15 mg/mL) was added into 1 mL of 0.73 M NaOH aqueous solution of DHTP (0.183 mmol) at 25°C. Then, 1 mL of aqueous solution of M(CH_3_COO)_2_·4H_2_O (0.183 mmol) (M = Ni, Co, Mg) and Zn(CH_3_COO)_2_·2H_2_O (0.183 mmol) was mixed with the solution, sonicated for 1 min and stirred for 6 h at 25°C. The precipitate was collected by centrifugation and washed with H_2_O and MeOH three times. Finally, the precipitate was soaked in MeOH for 48 h and dried under a vacuum at 60°C overnight.

### X-ray absorption spectra

The Au L_3_-edge XANES and EXAFS experiments were conducted at the 1W1B beamline station in the Beijing Synchrotron Radiation Facility (BSRF) and the BL14W1 beamline station in the Shanghai Synchrotron Radiation Facility (SSRF). All the data were collected in transmission mode. The incident beam was monochromatized using a Si (111) double-crystal monochromator and a harmonic rejection mirror (S4) was used to eliminate harmonics at high X-ray energy levels. Data reduction, analysis and EXAFS fitting were performed using the Athena and Artemis software packages. The energy calibration of the catalysts was conducted using a standard metal foil as a reference, which was measured simultaneously.

## Supplementary Material

nwae252_Supplemental_File
