# Supplementary material for: Regulating interaction with surface ligands on Au25 nanoclusters by multivariate metal–organic framework hosts for boosting catalysis
Source: Natl Sci Rev. 2024 Jul 23;11(10):nwae252. doi: 10.1093/nsr/nwae252 (PMC11409874; doi:10.1093/nsr/nwae252)
Supplement: nwae252_Supplemental_File [file nwae252_supplemental_file.doc]

**Supporting Information**

**Regulating Interaction with Surface Ligands on Au_25_ Nanoclusters by Multivariate Metal-Organic Framework Hosts for Boosting Catalysis**

He Wang^1,2,†^, Xiaokang Liu^3,†^, Yulong Zhao^1,2^, Zhihu Sun^3^, Yue Lin^1^, Tao Yao^3,*^ and Hai-Long Jiang^1,2,*^

^1^Hefei National Research Center for Physical Sciences at the Microscale, ^2^Department of Chemistry, University of Science and Technology of China, Hefei, Anhui 230026, P. R. China

^3^National Synchrotron Radiation Laboratory, University of Science and Technology of China, Hefei, Anhui 230029, P. R. China

†These authors contributed equally to this work.

*Corresponding author. Email: [yaot@ustc.edu.cn](mailto:yaot@ustc.edu.cn) (T.Y.); [jianglab@ustc.edu.cn](mailto:jianglab@ustc.edu.cn) (H.-L.J.)

**Section 1. Materials and Equipments**

All the chemicals were obtained from commercial sources and were not further purified otherwise stated. UV-vis spectra were obtained on a Shimadzu UV-2700 spectrophotometer. Powder X-ray diffraction (PXRD) patterns were recorded on a Rigaku Miniflex 600 instrument (Japan) equipped with graphite monochromatized Cu Kα radiation (λ = 1.54 Å). N_2_ sorption measurements were conducted at 77 K using a Micrometritics ASAP 2020. Scanning electron microscopy (SEM) was performed on a Zeiss Supra 40 scanning electron microscope at an acceleration voltage of 5 kV. High-angle annular dark-field scanning transmission electron microscopy (HAADF-STEM) and energy dispersive spectroscopy (EDS) mapping analyses were performed on a Talos F200X instrument equipped with Super-X EDX operating at 200 kV. Secondary electron scanning transmission electron microscopy (SE-STEM) and corresponding HAADF-STEM images were obtained with a JEOL JEM-F200 instrument. The Au and other transition metal contents were measured on a Thermo Scientific iCAP 7400 series instrument via inductively coupled plasma atomic emission spectrometry (ICP‒AES). X-ray photoelectron spectroscopy (XPS) measurements were recorded by using a Thermo ESCALAB 250 high-performance electron spectrometer with S2 monochromatized Al Kα as the excitation source. Electron paramagnetic resonance (EPR) spectra were measured by means of a JEOL JES-FA200 EPR spectrometer. In order to detect possible free radical signals in the solution, the substrate and catalyst were well dispersed by sonication in 1 mL of aqueous solution and tested at room temperature. Photoluminescence (PL) measurements were performed on a Hitachi FL-4600 fluorescence spectrometer. The catalyst was thoroughly dispersed in 2 mL of aqueous solution by sonication and the Au_25_ NCs content in the solution was controlled to be 0.05 mg, and tested under 420 nm excitation with a slit width of 10 nm. The catalytic reaction products were identified and quantified by gas chromatography (GC; Shimadzu 2010 Plus, 0.25 mm × 30 m Rtx-5 capillary column) and then determined using an Agilent Technologies model 7890A gas chromatograph and an Agilent Technologies model 5975C mass spectrometer as detectors.

**Section 2. Preparation of catalysts**

**Synthesis of Au_25_(Cys)_18_ NCs.** The Au_25_(Cys)_18_ was synthesized based on previous method with some modifications [1]. First, 15 mL aqueous solutions of HAuCl_4_·4H_2_O (10 mM) and 15 mL of Cys (20 mM) were mixed and stirred at 25 °C, leading to the formation of milky white Cys-Au(I) complexes. Then, 0.9 mL of aqueous solution of NaOH (1 M) was added to the reaction mixture, and the reaction mixture rapidly turned from milky white to clear. Next, 0.3 mL of NaBH_4_ solution was added (prepared by dissolving 21 mg of NaBH_4_ powder in 5 mL of 0.2 M NaOH solution). After stirring for 3 h, the Au_25_(Cys)_18_ products were collected, washed thoroughly with acetonitrile-water (v/v = 3:1) five times, and then dried under vacuum at room temperature to obtain brown-black powder.

**Synthesis of Zn-MOF-74.** The Zn-MOF-74 was synthesized at room temperature according to previous method with some modifications [2]. First, 1 mL of aqueous solution of Zn(CH_3_COO)_2_·2H_2_O (0.732 mmol) was added dropwise to 1 mL of 0.73 M NaOH aqueous solution of DHTP (0.183 mmol) with vigorous stirring. After stirring for 6 h at 25 °C, the precipitate was collected by centrifugation and washed with H_2_O and MeOH three times. Finally, the precipitate was soaked in MeOH for 48 h and dried under vacuum at 60 °C overnight.

**Synthesis of** **M-MOF-74 (M= Ni, Co, Mg).** The M-MOF-74 was synthesized at room temperature according to previous method with some modifications [2]. First, 1 mL of aqueous solution of mixed M(CH_3_COO)_2_·4H_2_O (0.183 mmol) (M = Ni, Co, Mg) and Zn(CH_3_COO)_2_·2H_2_O (0.183 mmol) was added dropwise into 1 mL of 0.73 M NaOH aqueous solution of DHTP (0.183 mmol) with vigorous stirring. After stirring for 6 h at 25 °C, the precipitate was collected by centrifugation and washed with H_2_O and MeOH three times. Finally, the precipitate was soaked in MeOH for 48 h and dried under vacuum at 60 °C overnight.

**Synthesis of** **Au_25_/Ni-MOF-74.** Typically, 100 mg of Ni-MOF-74 was dispersed in 5 mL of aqueous solution under sonication, followed by the addition of 0.2 mL of aqueous solution of Au_25_(Cys)_18_ (15 mg/mL). After stirring for 6 h, the solution was centrifuged. The dark green precipitate was washed with MeOH and dried under vacuum at 60 °C overnight.

**Synthesis of** **Au_NPs_-Cys.** The Au_NPs_-Cys was synthesized at room temperature according to previous method with some modifications [3]. First, Cys (120 mg) was mixed with 100 ml of NaOH aqueous solution (0.025 M), and 2 mL of HAuCl_4_·4H_2_O aqueous solution (50 mM) was added with vigorous stirring for 2 h at 25 °C. Then, 12.5 mL of NaBH_4_ cold aqueous solution (0.01 M) was added, and the pH was adjusted to 9 using 0.1 M HCl. After stirring for 2 h, the Au_NPs_-Cys products were collected, washed thoroughly with acetonitrile–water (v/v = 3:1) five times, and then dried under vacuum at room temperature to obtain brown‒black powder.

**Synthesis of** **Au_NPs_@Ni-MOF-74.** Typically, 0.2 mL of aqueous solution of Au_NPs_-Cys (15 mg/mL) was added to 1 mL of 0.73 M NaOH aqueous solution of DHTP (0.183 mmol) at 25 °C. Then, 1 mL of aqueous solution of Ni(CH_3_COO)_2_·4H_2_O (0.183 mmol) and Zn(CH_3_COO)_2_·2H_2_O (0.183 mmol) was mixed with the solution, sonicated for 1 min, and stirred for 6 h at 25 °C. The precipitate was collected by centrifugation and washed with H_2_O and MeOH three times. Finally, the precipitate was soaked in MeOH for 48 h and dried under vacuum at 60 °C overnight.

**Section 3. Catalytic Reactions**

**Intramolecular cascade reaction of 2-nitrobenzonitrile.** Typically, 10 mg of catalyst powder (0.3 mg of Au_25_(Cys)_18_) was dispersed in 4 mL of H_2_O, followed by the addition of 0.1 mmol of 2-nitrobenzonitrile. Subsequently, 2 mL of NaBH_4_ aqueous solution (4 mg/mL) was added, and the mixture was stirred (300 rpm) at 25 °C. After the completion of the reaction, the reaction mixture was centrifuged to separate the catalyst from the solvent. The conversion and selectivity were subsequently quantified by GC, and the products were confirmed by GC-MS.

**Recyclability test of Au_25_@Ni-MOF-74.** The catalyst was collected from the reaction mixture by centrifugation, washed three times with MeOH, and dried under vacuum after each cycle. Afterward, the process was repeated for the next cycle under the same reaction conditions.

**Intramolecular** **cascade reaction with other substrates.** Typically, 10 mg of catalyst powder (0.3 mg of Au_25_(Cys)_18_) was dispersed in 4 mL of H_2_O, followed by the addition of 0.1 mmol of the other substrate, for example, nitrobenzene, benzonitrile, anthranilonitrile, 2-nitrobenzamide, 2-nitrobenzaldehyde, 2-cyanobenzaldehyde, 4-nitrobenzonitrile or 4-cyanobenzaldehyde. Then, 2 mL of NaBH_4_ aqueous solution (4 mg/mL) was added, and the mixture was stirred (300 rpm) at 25 °C. After the reaction, the reaction mixture was centrifuged to separate the catalyst from the solvent. The conversion and selectivity were subsequently quantified by GC, and the products were confirmed by GC-MS.


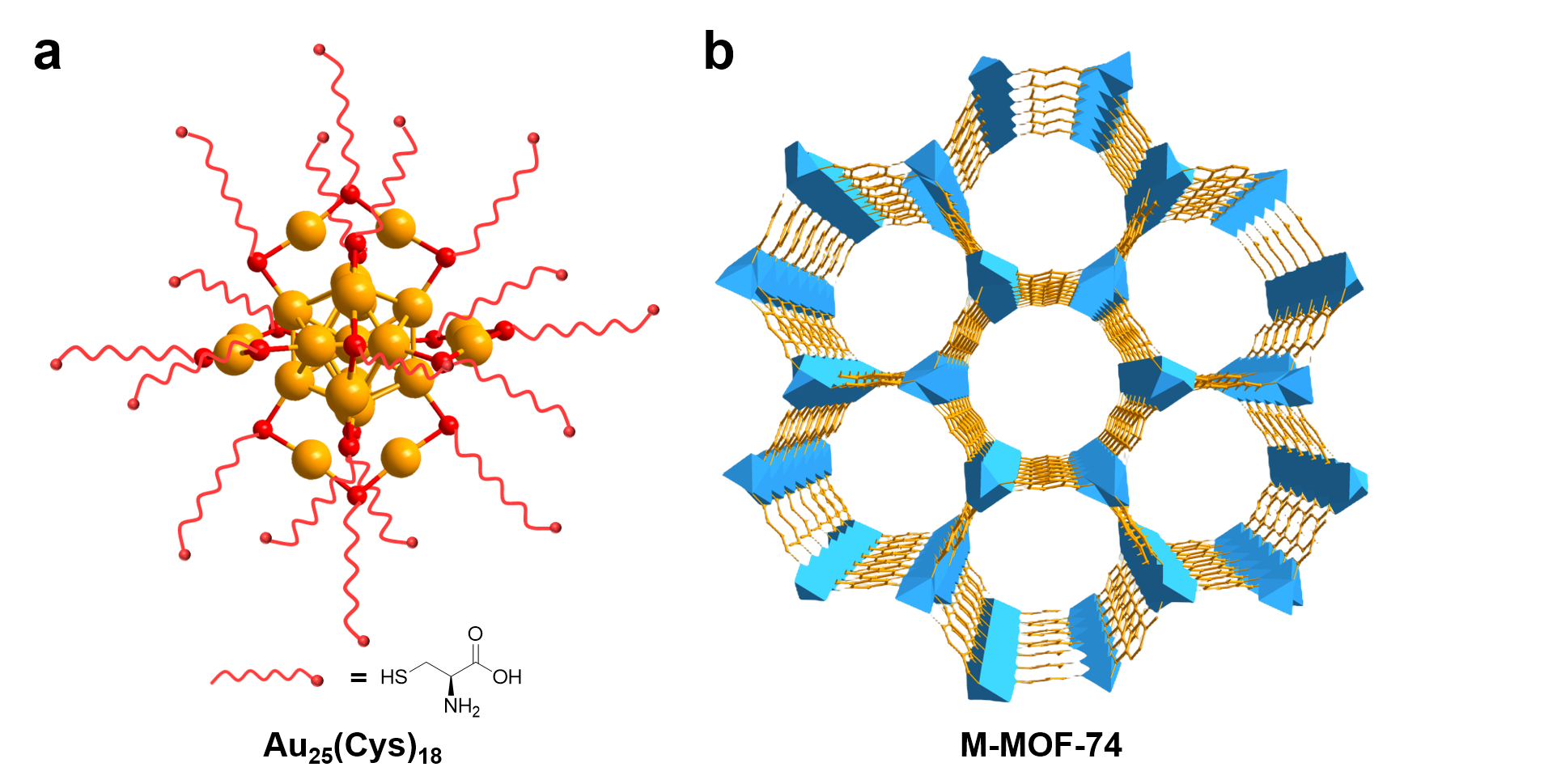


**Supplementary Figure S1.** The schematic illustration of structures of (a) Au_25_(Cys)_18_ and (b) M-MOF-74.

**Supplementary Figure S2.** (a) UV-vis spectrum of Au_25_(Cys)_18_ in water. (b) HAADF-STEM image of the as-synthesized Au_25_(Cys)_18_ NCs.

Fingerprint absorption peaks can be observed at 400 nm, 450 nm, 670 nm, and 780 nm in Supplementary Figure S1, in agreement with those in the previous literature [1].

**Supplementary Figure S3.** (a) Powder XRD patterns of simulated MOF-74 and as-synthesized M-MOF-74. (b) Powder XRD patterns of simulated MOF-74, as-synthesized Au_25_@M-MOF-74 and Au_25_/Ni-MOF-74.

**Supplementary Figure S4.** (a) N_2_ sorption isotherms of Au_25_@M-MOF-74 and Au_25_/Ni-MOF-74 at 77 K and (b) the corresponding pore size distributions based on the DFT model.

**Supplementary Figure S5.** (a) UV‒vis spectra of Au_25_(Cys)_18_ and Au_25_(Cys)_18_ detached from Au_25_@M-MOF-74. (b) UV‒vis spectra of M-MOF-74.

The Au_25_(Cys)_18_ NCs detected from Au_25_@M-MOF-74 display the characteristic bands of as-synthesized Au_25_(Cys)_18_ NCs at ~450 nm and ~670 nm and the UV-vis spectra of M-MOF-74 are quite different from that of Au_25_(Cys)_18_ NCs. Therefore, it can be ensured that the UV-vis spectra after Au_25_@MOF-74 cleavage are attributed to the Au_25_(Cys)_18_ NCs.

**Supplementary Figure S6.** SEM images of the as-synthesized (a) Au_25_/Ni-MOF-74, (b) Au_25_@Co-MOF-74, (c) Au_25_@Zn-MOF-74 and (d) Au_25_@Mg-MOF-74.

**Supplementary Figure S7.** (a) HAADF-STEM and (b) SE-STEM images of Au_25_/Ni-MOF-74.

By directly comparing the HAADF-STEM and SE-STEM images acquired at the same location, it becomes evident that Au_25_(Cys)_18_ NCs within Au_25_(Cys)_18_/Ni-MOF-74, as observed in the HAADF-STEM image (Supplementary Fig. S6a), are distinctly discernible at nearly identical positions in the SE-STEM image (Supplementary Fig. S6b). These findings clearly demonstrate that the majority of the Au_25_(Cys)_18_ NCs are deposited on the external surface of the MOF in Au_25_(Cys)_18_/Ni-MOF-74.


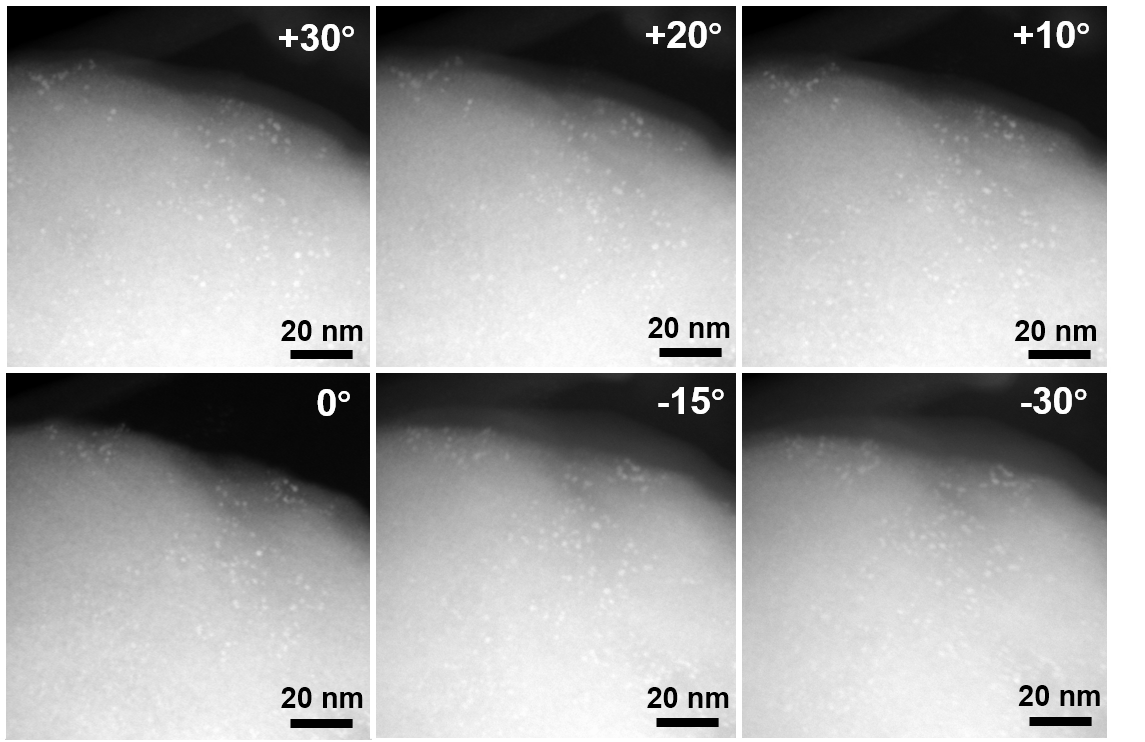


**Supplementary Figure S8.** HAADF-STEM images of Au_25_@Ni-MOF-74 projected at different tilt axis angles from +30° to −30°.

**Supplementary Figure S9.** HAADF-STEM image of (a) Au_25_/Ni-MOF-74 and the corresponding EDS elemental mapping for (b) Au, (c) Ni, (d) Zn and (e) their overlap.

**Supplementary Figure S10.** HAADF-STEM image of (a) Au_25_@Zn-MOF-74 and the corresponding EDS elemental mapping for (b) Au, (c) S, (d) Zn and (e) their overlap.

**Supplementary Figure S11.** HAADF-STEM image of (a) Au_25_@Co-MOF-74 and the corresponding EDS elemental mapping for (b) Au, (c) Co, (d) Zn and (e) their overlap.

**Supplementary Figure S12.** HAADF-STEM image of (a) Au_25_@Mg-MOF-74 and the corresponding EDS elemental mapping for (b) Au, (c) Mg, (d) Zn and (e) their overlap.

**Supplementary Figure S13.** (a) UV-vis spectra of as-prepared Au_25_(Cys)_18_ and Au_25_(Cys)_18_ after the catalytic reaction. (b) HAADF-STEM image of Au_25_(Cys)_18_ NCs after the catalytic reaction.

**Supplementary Figure S14.** Powder XRD patterns of simulated MOF-74 and Au_25_@M-MOF-74 after the catalytic reaction.

**Supplementary Figure S15.** (a) UV-vis spectrum of Au NPs in water. (b) HAADF-STEM image of Au NPs with the protection of Cys.

**Supplementary Figure S16.** (a) Powder XRD patterns of simulated MOF-74 and as-synthesized Au_NPs_@Ni-MOF-74. (b) HAADF-STEM image of Au_NPs_@Ni-MOF-74.

**Supplementary Figure S17.** Three catalytic cycles of Au_25_@Ni-MOF-74 and Au_25_/Ni-MOF-74.

**Supplementary Figure S18.** HAADF-STEM images of (a) Au_25_@Ni-MOF-74 and (b) Au_25_/Ni-MOF-74 after three cycles of catalytic reactions.

**Supplementary Figure S19.** Powder XRD patterns of as-synthesized Au_25_@Ni-MOF-74 and the Au_25_@Ni-MOF-74 after five runs of catalytic recycling.

**Supplementary Figure S20.** HAADF-STEM image of (a) Au_25_@Ni-MOF-74 after five runs of catalytic recycling and the corresponding EDS elemental mapping for (b) Au, (c) Ni, (d) Zn, and (e) their overlap.

**Supplementary Figure S21.** Hot filtration test of Au_25_@Ni-MOF-74 after 30 min of reaction. Reaction conditions: 0.1 mmol substrate, 6 mL H_2_O, 8 mg NaBH_4_, 10 mg catalyst, 25 °C.

Upon hot filtration, the yield does not increase, indicating the absence of active site leaching and truly heterogeneous catalysis.

**Supplementary Figure S22.** (a) The single-electron transfer process of Au_25_(Cys)_18_ with a negative charge (abbreviated as Au_25_^-^) in the intramolecular cascade reaction of 2-nitrobenzonitrile. (b) UV-vis spectra of single electron transfer processes in the catalysis of Au_25_^-^.

**Supplementary Figure S23.** EPR signals of Au_25_@Ni-MOF-74 and the mixture of Ni-MOF-74, Au_25_(Cys)_18_, or Au_25_@Ni-MOF-74 with the 2-nitrobenzonitrile as substrate.

**Supplementary Figure S24.** Mass spectra of the intramolecular cascade reaction of 2-nitrobenzonitrile products under different deuterated reaction conditions.

**Supplementary Figure S25.** Proposed mechanism of the intramolecular cascade reaction of 2-nitrobenzonitrile over Au_25_@M-MOF-74.

The mechanism is proposed according to the previous report [4]. Specifically, Au_25_(Cys)_18_ serves as an electron mediator, initiating a single-electron transfer from Au_25_(Cys)_18_ to 2-nitrobenzonitrile. Subsequently, the resulting free N radical undergoes conversion into an intermediate molecule known as 2-(hydroxyamino) benzonitrile by reaction with active hydrogen species and subsequently loses one molecule of H_2_O. Following this step, an intramolecular coupling reaction takes place, forming a five-membered heterocyclic intermediate. This intermediate then captures another electron from Au_25_(Cys)_18_ to interact with active hydrogen species, ultimately yielding the final reduced product. The process involves the supplementation of Au_25_(Cys)_18_ with the single electron from NaBH_4_, which in turn leads to the breaking of B-H and O-H bonds in NaBH_4_ and H_2_O at the Au_25_(Cys)_18_ interface, generating reactive hydrogen species necessary for reducing the substrate.

**Supplementary Figure S26.** The different XAFS spectra of Au_25_@M-MOF-74 with Au_25_(Cys)_18_.

**Supplementary Figure S27.** EXAFS k^2^χ(k) space spectra of Au_25_(Cys)_18_, Au_25_@M-MOF-74, and the Au_25_@Ni-MOF-74 after catalysis.

**Supplementary Figure S28.** The bond lengths of Au-S are extracted from the refinement of the FT-EXAFS spectra of Au_25_(Cys)_18_ and Au_25_@M-MOF-74.

**Supplementary Figure S29.** (a) Au L_3_-edge XANES spectra of Au_25_(Cys)_18_, Au_25_@Ni-MOF-74 and the Au_25_@Ni-MOF-74 after catalysis. (b) Au L_3_-edge FT-EXAFS spectra of Au_25_(Cys)_18_, Au_25_@Ni-MOF-74, and the Au_25_@Ni-MOF-74 after catalysis.

**Supplementary Table S1.** The measured different metal contents in the catalysts.a

| **Entry** | **Sample** | **Au content (wt%)** | **Zn content (wt%)** | **Ni/Co/Mg content (wt%)** | **molar ratio of Zn : M (M = Ni, Co, Mg)** |
| --- | --- | --- | --- | --- | --- |
| 1 | Au_25_@Zn-MOF-74 | 2.0 | 34.6 | N.D.^b^ | - |
| 2 | Au_25_@Ni-MOF-74 | 2.1 | 16.8 | 17.4 | 1.0 |
| 3 | Au_25_@Co-MOF-74 | 2.0 | 16.5 | 17.2 | 0.9 |
| 4 | Au_25_@Mg-MOF-74 | 2.1 | 21.8 | 8.8 | 1.0 |
| 5 | Au_25_/Ni-MOF-74 | 2.0 | 17.2 | 17.7 | 1.1 |
| 6 | Au_25_@Ni-MOF-74  (after catalysis) | 2.1 | 16.6 | 17.5 | 1.0 |
| 7 | Au_25_/Ni-MOF-74  (after catalysis) | 1.8 | 17.0 | 17.4 | 1.1 |
| 8 | Au_NPs_@Ni-MOF-74 | 2.2 | 16.5 | 16.8 | 1.1 |

^a^The data are based on inductively coupled plasma atomic emission spectroscopy (ICP-AES) results. ^b^N.D.: not detectable.

**Supplementary Table S2.** The intramolecular cascade reaction of 2-nitrobenzonitrile over control catalysts.^a^

| **Entry** | **Catalyst** | **Conv. (%)** | **Sel. (%)** |
| --- | --- | --- | --- |
| 1 | none | N.D.^b^ | - |
| 2 | Zn-MOF-74 | N.D. | - |
| 3 | Ni-MOF-74 | N.D. | - |
| 4 | Co-MOF-74 | N.D. | - |
| 5 | Mg-MOF-74 | N.D. | - |
| 6 | Au_25_(Cys)_18_ | 20.6 | 91.0 |
| 7 | Au_25_(Cys)_18_ + Zn-MOF-74 | 21.1 | 91.2 |
| 8 | Au_25_(Cys)_18_ + Ni-MOF-74 | 21.4 | 91.5 |
| 9 | Au_25_(Cys)_18_ + Co-MOF-74 | 21.0 | 91.2 |
| 10 | Au_25_(Cys)_18_ + Mg-MOF-74 | 20.8 | 91.0 |
| 11 | Au_NPs_@Ni-MOF-74 | 15.2 | 89.5 |

^a^Reaction conditions: 10 mg of cat. or 0.3 mg of Au_25_(Cys)_18_, 0.1 mmol of substrate, 6 mL of H_2_O, 8 mg of NaBH_4_, room temperature; ^b^N.D.: not detectable.

**Supplementary Table S3.** Catalytic reduction reactions of various substrates over Au25@Ni-MOF-74.a

| **Entry** | **Substrate** | **Product** | **Time (h)** | **Conv. (%)** | **Sel. (%)** |
| --- | --- | --- | --- | --- | --- |
| 1 |  |  | 24 | 65.8 | 92.5 |
| 2 |  | N.D.^b^ | 24 | N.D. | N.D. |
| 3 |  | N.D. | 24 | N.D. | N.D. |
| 4 |  | N.D. | 24 | N.D. | N.D. |
| 5 |  |  | 1 | 99.9 | 99.9 |
| 6 |  |  | 1 | 98.5 | 99.0 |
| 7 |  |  | 24 | 95.6 | 96.2 |
| 8 |  |  | 24 | 90.4 | 98.5 |

^a^Reaction conditions: 10 mg cat. (~2 wt% Au loading), 0.1 mmol substrate, 6 mL H_2_O, 8 mg NaBH_4_, room temperature; ^b^N.D.: not detectable.

**Supplementary Table S4.** Structural parameters obtained by curve-fitting analysis of EXAFS data.

| **Sample** | **Path** | **CN^a^** | **R(Å)^b^** | **σ^2^(10^-3^Å^2^)^c^** | **ΔE_0_ (eV)^d^** | **R-factor^e^** |
| --- | --- | --- | --- | --- | --- | --- |
| Au_25_ | Au-S | 1.0±0.1 | 2.31±0.01 | 3.8±0.8 | 6.2±1.1 | 0.012 |
|  | Au-Au1 | 1.2±0.2 | 2.69±0.02 | 7.7±1.0 | 5.9±1.3 |  |
|  | Au-Au2 | 1.3±0.3 | 2.77±0.02 | 9.2±1.2 | 5.9±1.3 |  |
| Au_25_@Ni-MOF-74 | Au-S | 1.6±0.1 | 2.31±0.01 | 3.8±0.8 | 8.8±1.1 | 0.003 |
|  | Au-Au1 | 1.2±0.2 | 2.73±0.02 | 6.9±1.4 | 9.3±1.6 |  |
|  | Au-Au2 | 1.6±0.5 | 2.88±0.02 | 9.5±2.0 | 9.3±1.1 |  |
| Au_25_@Co-MOF-74 | Au-S | 1.5±0.1 | 2.31±0.01 | 3.8±0.8 | 8.0±1.5 | 0.008 |
|  | Au-Au1 | 1.2±0.2 | 2.71±0.01 | 6.6±1.9 | 8.5±1.8 |  |
|  | Au-Au2 | 1.6±0.6 | 2.86±0.02 | 9.5±3.3 | 8.5±1.8 |  |
| Au_25_@Zn-MOF-74 | Au-S | 1.6±0.1 | 2.30±0.01 | 3.8±0.8 | 7.0±1.2 | 0.011 |
|  | Au-Au1 | 1.0±0.1 | 2.70±0.02 | 8.5±3.0 | 6.5±2.6 |  |
|  | Au-Au2 | 1.5±0.5 | 2.76±0.02 | 9.3±1.5 | 6.5±2.6 |  |
| Au_25_@Mg-MOF-74 | Au-S | 1.5±0.1 | 2.31±0.01 | 3.8±0.8 | 8.0±1.1 | 0.014 |
|  | Au-Au1 | 1.2±0.1 | 2.68±0.01 | 8.2±1.9 | 5.9±2.2 |  |
|  | Au-Au2 | 1.4±0.3 | 2.76±0.02 | 11.0±2.0 | 5.9±2.2 |  |
| Au_25_@Ni-MOF-74 after  catalysis | Au-S | 1.5±0.1 | 2.31±0.01 | 3.8±0.8 | 5.2±0.8 | 0.010 |
|  | Au-Au1 | 1.2±0.1 | 2.70±0.02 | 7.6±2.3 | 9.0±1.8 |  |
|  | Au-Au2 | 1.5±0.5 | 2.87±0.02 | 10.2±4.4 | 9.0±2.4 |  |

^a^CN: coordination numbers; ^b^R: bond lengths; ^c^σ^2^: Debye-Waller factors; ^d^ΔE_0_: the inner potential correction; ^e^R-factor: goodness of fit.

**References**

1. Yuan X, Zhang B and Luo Z *et al*. Balancing the Rate of Cluster Growth and Etching for Gram‐Scale Synthesis of Thiolate‐Protected Au_25_ Nanoclusters with Atomic Precision. *Angew Chem Int Ed* 2014; **53**: 4623-7.
2. Garzón-Tovar L, Carné-Sánchez A and Carbonell C *et al*. Optimised room temperature, water-based synthesis of CPO-27-M metal-organic frameworks with high space-time yields. *J Mater Chem A* 2015; **3**: 20819-26.
3. Cappellari PS, Buceta D and Morales GM *et al*. Synthesis of Ultrasmall Cysteine-capped Gold Nanoparticles by pH Switching of the Au(I)–cysteine Polymer. *J Colloid Interface Sci* 2015; **441**: 17-24.
4. Chong H, Li P and Wang S *et al.* Au_25_ Clusters as Electron-Transfer Catalysts Induced the Intramolecular Cascade Reaction of 2-nitrobenzonitrile. *Sci Rep* 2013; **3**: 3214.
